# Pathogenic implications, incidence, and outcomes of COVID-19 in autoimmune inflammatory joint diseases and autoinflammatory disorders

**DOI:** 10.1186/s42358-021-00204-5

**Published:** 2021-07-08

**Authors:** Piero Ruscitti, Alessandro Conforti, Paola Cipriani, Roberto Giacomelli, Marco Tasso, Luisa Costa, Francesco Caso

**Affiliations:** 1 Rheumatology Unit, Department of Biotechnological and Applied Clinical Sciences, University of L’Aquila, L’Aquila, Italy; 2 Rheumatology and Immunology Unit, Department of Medicine, University of Rome ‘Campus Biomedico’, Rome, Italy; 3 Rheumatology Unit, Department of Clinical Medicine and Surgery, School of Medicine, University of Naples Federico II, Naples, Italy

**Keywords:** Rheumatoid arthritis, Spondiloarthritis, Psoriatic arthritis, COVID-19, SARS-COV-2

## Abstract

As the coronavirus disease 2019 (COVID-19) pandemic caused by severe acute respiratory syndrome coronavirus 2 (SARS-CoV-2) continues to spread rapidly, there are still many unresolved questions of how this virus would impact on autoimmune inflammatory joint diseases and autoinflammatory disorders. The main aim of this paper is to describe the main studies focusing their attention on COVID-19 incidence and outcomes of rheumatoid arthritis (RA), spondylarthritis (SpA), and autoinflammatory disease cohorts. We also revised possible pathogenic mechanisms associated with. Available data suggest that, in patients with RA and SpA, the immunosuppressive therapy, older age, male sex, and the presence of comorbidities (hypertension, lung disease, diabetes, CVD, and chronic renal insufficiency/end-stage renal disease) could be associated with an increased risk of infections and high rate of hospitalization. Other studies have shown that lower odds of hospitalization were associated with bDMARD or tsDMARDs monotherapy, driven largely by anti-TNF therapies. For autoinflammatory diseases, considering the possibility that COVID-19 could be associated with a cytokine storm syndrome, the question of the susceptibility and severity of SARS-CoV-2 infection in patients displaying innate immunity disorders has been raised. In this context, data are very scarce and studies available did not clarify if having an autoinflammatory disorder could be or not a risk factor to develop a more severe COVID-19. Taking together these observations, further studies are likely to be needed to fully characterize these specific patient groups and associated SARS-CoV-2 infection.

## Introduction

As the coronavirus disease 2019 (COVID-19) pandemic caused by severe acute respiratory syndrome coronavirus 2 (SARS-CoV-2) continues to spread rapidly, there are still many unresolved questions of how this virus would impact on rheumatic musculoskeletal diseases (RMDs) [1–3]. Evidence have highlighted that mechanisms underlining autoimmune and autoinflammatory conditions are critically involved in the host defense and response versus SARS-COV-2, and in more severe cases, leading to a cytokine storm syndrome (CSS), with an acute respiratory distress syndrome (ARDS), multiorgan failure and death [4–6]. Not at last, several studies have been conducted and are ongoing to evaluate the efficacy of biologic disease modifying antirheumatic drugs (bDMARDs) and targeted synthetic DMARDs (tsDMARDs) to treat COVID-19 CSS [7, 8]. Of note, among those, there are agents targeting interleukin-6 (IL-6) and IL-1, and Janus Kinase Inhibitors (JAKis), currently used for the treatment of inflammatory arthropathies and autoinflammatory disorders. However, due to the heterogeneity of randomized controlled trials (RCTs) design and cohorts, and the presence of limitations, as for example concomitant steroidal treatment, studies have showed mixed results on the impact of these therapies on COVID-19 outcomes [7, 8]. Many questions remain still open and one of the most important is whether patients with autoimmune arthropathies and autoinflammatory disorders may have different responses to the SARS-CoV-2 infection, because of impaired immunological and genetic background and/or concomitant antirheumatic treatment [9–11].

Until today, scientific literature has described high incidence and severe outcomes of COVID-19 in middle-aged and elderly subjects and/or patients with underlining diseases, such as diabetes, cardiovascular disease, chronic obstructive pulmonary disease (COPD) [12, 13]. Whether patients with RMDs, especially those receiving Disease Modifying Antirheumatic drugs (DMARDs), are at an increased risk of SARS- CoV-2 infection or severe COVID-19 disease remains to be clarified. On the other hand, it is well known that viral infection susceptibility and clinical aspects may be influenced both by inflammatory diseases, as well as immunomodulant and immunosuppressive therapies [14–16]. For this last point, several studies highlighted that patients with immunoinflammatory diseases on biologic (bDMARDs) may be at higher risk to test positive for COVID-19 and hospitalized, but with a not increased risk of Intensive Care Units (ICU) admission or death in comparison with general population. Hence, the COVID-19 pandemic has represented a hard challenge for Rheumatologists [17, 18].

Today, most studies on incidence and clinical outcomes are available. Thus, the aims of this paper are to describe the main studies focusing their attention on COVID-19 incidence and outcomes of rheumatoid arthritis (RA), spondylarthritis (SpA), and autoinflammatory disease cohorts. We also revised possible pathogenic mechanisms associated with.

## Methods

We designed a comprehensive research of literature on incidence and outcomes of COVID-19 during RMDs and autoinflammatory disorders by a review of reports published in international journals. We searched relevant English-language articles in MEDLINE (via Pubmed) until up February 2021, with the research terms: (“SARSCoV2” OR “COVID-19”) AND (“RMDs” OR “autoinflammatory disorders”). We also revised possible pathogenic mechanisms involved with. In preparing this work, we followed the proposed guidelines for narrative review [19]. Furthermore, we hand-searched reference lists of the relevant articles to find possible additional studies. Out of retrieved 417 articles, both observational studies and case series were assessed if reporting data about COVID-19 incidence and outcomes in patients with RA, SpA, or autoinflammatory diseases.

### SARS-CoV-2 infection and immune response

**Fig. 1:**
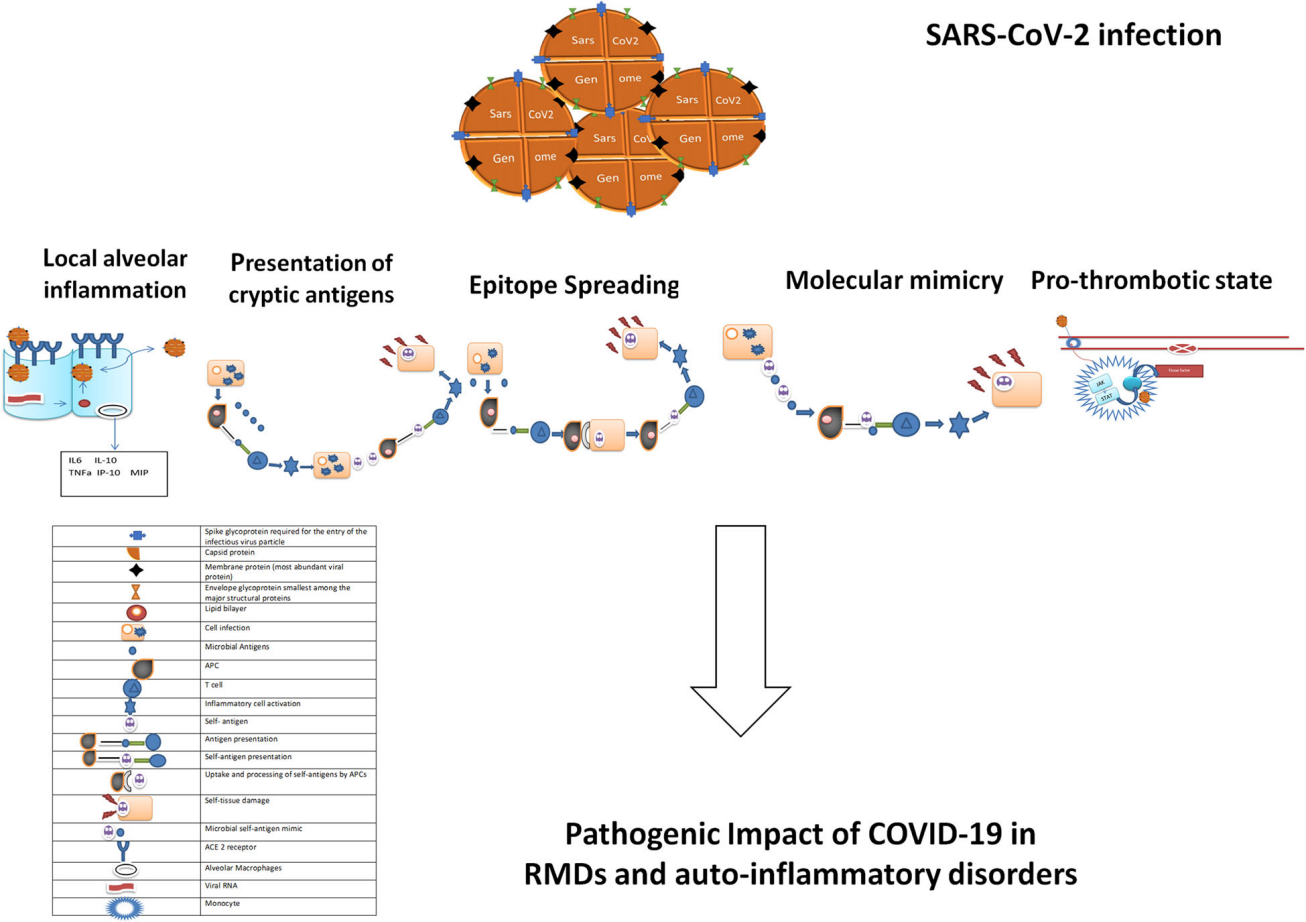
The figure describes the structure of SARS-CoV2 and the possible infection associated mechanism of autoimmunity. SarsCov2 infects epitelial cells by receptors angiotensin converting enzyme 2 (ACE2) resulting in replication and release of the virus with consequent pyroptosis and the release of damage associated molecular patterns. This process leads to local inflammation and secretion by alveolar macrophages of pro-inflammatory cytokines such as IL-6, IL-10, macrophages inflammatory protein 1 alfa (MIPa), and TNF. Furthermore, this figure describes epitope spreading where persistent infection leads to the uptake of microbial antigens by antigen presenting cells (APCs) and antigen presentation of T-cells. This mechanism carries an inflammatory cascade resulting in a tissue damage, a possible uptake of self-antigens by APC and subsequent presentation of these self-antigens to Tc ells causing an anti-self-reaction. After microbial infection self-antigens could be recognised and processed by APC; subsequently cryptic epitopes could be exposed and presented to self-reactive T-cells leading to an autoimmune response. This figure also describes the mechanism of molecular mimicry where microbial antigens could share antigenic similarly with self-antigens. The presentation of these self-antigen mimics by APC to cross reactive cells leads to an inflammatory response because T cells recognize both the microbial mimic and its respective self-antigens. Finally, viral particles and proinflammatory cytokines induce the activation of blood monocytes which respond by inducing tissue factor membrane expression contributing to a pro-thrombotic state

The host immune response has a crucial role for the resolution of COVID-19, but, at the same time, it is implicated in the occurrence of major clinical manifestations of the disease, mostly in more severe patients, as shown in Fig. 1 [20]. Thus, SARS-CoV-2 infection is an example of environmental factor which could trigger an aberrant inflammatory reaction [20]. As for previous evidence, it is well known that an infective trigger may exacerbate an inflammatory disease [21]. In fact, a persistent infection may result in the uptake of microbial antigens by antigen presenting cells (APCs) and presentation of these antigens to microbe-specific T cells [21]. The consequent activation of the pro-inflammatory cascade in a persistently infected site may lead to a tissue damage and uptake of self-antigens by APCs [21]. The presentation of these self-antigens to self-reactive T cells may induce an immune reaction against self-tissues, spreading from a one against microbial antigen epitopes to some non-cross-reactive self-epitopes [21]. This mechanism leading to a reaction against self-antigens is recognized as epitope spreading. Simultaneously, a molecular mimicry may occur. This is a step related to a sufficient similarity between the amino acid sequences of the pathogen and some of the host antigens producing a reaction directed against the host [21]. Furthermore, the pro-inflammatory process during an infection may enhance the production of proteases and the processing of self-antigens by APCs. Additionally, subdominant cryptic antigens, which are unnoticed by the immune system under normal conditions, may be implicated in this process. Infections may also result in the development of pro-inflammatory disorders by driving the APCs to process and display such cryptic antigens [21].

SARS-CoV-2 infection may cause a significant reduction in circulating T cell subsets [22]. The specific depletion of CD4+T cells may lead to an enhanced immune-mediated interstitial pneumonitis and delayed clearance of SARS-CoV in the lungs [22]. In fact, in the pulmonary *interstitium* of SARS-CoV-infected patients, CD8+T cells are the most common infiltrative inflammatory cells, playing an important role in virus clearance as well as in immune-mediated injury [22]. In addition, SARS-CoV2 infection may activate lymphocytes and macrophage by the binding with angiotensin-converting enzyme 2 (ACE-2) receptor [23]. During COVID-19, it has been shown that ACE2-expressing macrophages contained SARS-CoV-2 nucleoprotein antigen. These cells also showed an upregulation of IL-6, which may contribute to the excessive inflammatory burden and correlate with the disease severity [23]. Moreover, other pro-inflammatory chemokines and pro-inflammatory cytokines may be secreted by SARS-CoV-2-infected alveolar macrophages, which may further enhance the inflammation [23]. Interestingly, during COVD-19, two chemokines (CCL2 and CCL7) resulted to be increased in the bronchoalveolar fluid [24]. Thus, the recruitment of CC-chemokine receptor 2-positive (CCR2+) pro-inflammatory monocytes is favored in COVID-19 with an associated depletion of tissue-resident alveolar macrophages [24]. These pro-inflammatory macrophages have been described to be enriched in genes associated with tissue repair and fibrosis, possibly leading to the fibrotic complications in these patients under mechanical ventilation [24]. In addition, a massive expansion of CD8+T cells with a tissue-resident memory T cell gene signature has been observed in bronchoalveolar fluid patients with mild COVID-19, associated with a minimal infiltration of pro-inflammatory monocytes [24]. This finding suggested that some pre-existing populations of tissue-resident memory T cells, with a possible cross-reactivity against SARS-CoV-2, may limit the activation of pathological macrophages, thus resulting in a protection against a more severe pro-inflammatory process. These observations paralleled with infections due to other highly pathogenic coronaviruses, such as SARS-CoV and MERS-CoV [24, 25]. In fact, an extensive cellular infiltration dominated by macrophages was found in post-mortem lungs from these patients [24, 25]. Similarly, high levels of interferon-γ (IFN-γ), IL-6, IL-12, transforming growth factor-β (TGF-β), CCL2, CXCL10, CXCL9, and IL-8 were reported in patients with SARS-CoV [25, 26]. Probably, during these disease, high rates of viral replication may lead to host cell cytolysis and the production of pro-inflammatory cytokines and chemokines by infected epithelial cells [26]. In addition, a delayed induction of antiviral interferon responses, owing to specific virus escape mechanisms, could promote the accumulation of pro-inflammatory cells in the lungs. This finding could be probably related to viral structural and non-structural proteins antagonizing IFN responses [26]. The antagonism could occur at various stages of the IFN signaling pathway: (1) preventing the pattern-recognition receptors (PRRs) recognition of viral; (2) impairing the PRR signaling through TBK1/inhibitor of nuclear factor-κB kinase subunit-ε (IKKε), TRAF3 and IRF3; (3) inhibiting the downstream of IFN signaling through STAT1; (4) promoting the host mRNA degradation and inhibiting host protein translation [26]. Furthermore, SARS-COV 2 replication in airway epithelial cells could cause high levels of virus-linked pyroptosis with associated vascular leakage [26]. This could also be a trigger for the subsequent pro-inflammatory response [26]. By PRRs, alveolar epithelial cells and alveolar macrophages may detect the released pathogen-associated molecular patterns (PAMPs), such as viral RNAs, and damage-associated molecular patterns (DAMPs) [26]. This may lead to a local inflammation resulting in an increased secretion of the pro-inflammatory cytokines and chemokines IL-6, IFN-γ, MCP1, and IP-10 into the blood of COVID-19 patients [26]. In addition, the pulmonary recruitment of immune cells from the blood and the infiltration of lymphocytes into the airways may explain the lymphopenia and an increased neutrophil–lymphocyte ratio observed in patients with SARS-CoV-2 infection [26]. Finally, some studies reported the development of autoantibodies in patients with COVID-19, particularly anti-cardiolipin, anti-β2-glycoprotein, and antinuclear antibodies [27, 28]. An increase of ACPA was reported after SARS-CoV2 infection suggesting an epitope spreading before the clinical onset of arthritis [27, 28]. Additional studies reported that the onset of anti-cardiolipin, anti-β2-glycoprotein, and lupus anticoagulant could be associated with the thromboembolic complications occurring in COVID-19 [29].

### Risk of infections in RMDs and autoinflammatory disorders

Patients with RMDs are recognized to have an increased risk of infections related to disease activity, comorbidities, and immunomodulatory therapy [30]. This is due to a combination of underlying disease characteristics and treatment-related factors. In fact, it must be pointed out that inflammatory diseases may also be associated with some immunological dysfunctions increasing the risk of infections [30]. In addition, both csDMARDs and bDMARDs are associated with a higher risk for serious infections [30]. These drugs, targeting key molecules involved in the immune response against infectious antigens, may consequently increase the susceptibility to viruses and bacteria. At the same time, these therapies could provide substantial benefit for controlling RMDs and limiting long-term disability and improving the quality of life [30].

In this context, it is well-known that glucocorticoids (GCs) usage is associated with an enhanced risk of infections, which is correlated with dosages and time of treatment. In fact, a prolonged therapy, even if at low dosages, may enhance the risk of serious infections, which is further increased by the concomitant administration of csDMARDs [31, 32]. Among these drugs, methotrexate (MTX) is the most common administered in RMDs and it is also associated with an increased risk of infection [33, 34]. In fact, recently a meta-analysis showed that MTX is associated with a higher risk of all infections, mainly in RA [35].

In addition, TNFis are associated with an increased risk of serious infections, since the inhibition of this molecule may impair its regulation of immune cell proliferation, differentiation, and survival [36]. In fact, it has been shown that TNF knockout mice rapidly succumb to tuberculosis infection [36]. National registries, open-label extension studies, and retrospective cohorts showed that TNF inhibition may enhance the risk of tuberculosis in patients with RMD [36–39] TNFi could also influence the odds of clearance or reactivation of chronic hepatitis B virus infection [40]. Furthermore, TNFi may increase the risk of fungal infections, such as cryptococcosis, aspergillosis, and pneumocystis jirovecii pneumonia [36, 38].

IL-6-targeted agents are largely used in the treatment of patients with RMDs and autoinflammatory disorders [40–43]. The inhibition of this cytokine, whose signaling pathway uses the protein gp130 as a common signal transducer, could impair the host capacity for generating acute phase responses, including Th17 differentiation and long-lived plasma cell generation [36, 42, 43]. In fact, some clinical data derived from RCTs supports the notion that IL-6-targeting agents increase the risk of infection to a degree similar to TNFis [36, 40, 52, 53]. On the other hand, neutropenia induced by IL-6 inhibition would frequently occur, due to a margination of neutrophils rather than peripheral sequestration. This finding could increase the risk of infection following this therapeutic strategy [40, 41].

Different IL-17-targeted agents have been recently approved for patients with PsA and SpA. IL-17 stimulates neutrophil granulopoiesis and chemotaxis, expression of antimicrobial peptides (β-defensin-2) [41–44]. Thus, patients with RMDs treated with IL-17-targeted agents may be exposed to an increased risk of mucocutaneous candidiasis [42–44]. No cases of active tuberculosis have been reported in a review of clinical trial data [37, 42, 43, 45].

Abatacept is a fusion protein designed to interfere with the binding of CD80/86 molecules on antigen presenting cells/dendritic cells with their CD28 receptors on the surface of CD4 T cells, used in the treatment of RA. Although lower than TNFis, the risk of infection-related hospitalizations is further recognized in patients treated with abatacept [36, 43, 46].

Furthermore, rituximab (RTX), a chimeric monoclonal antibody against the B-cell specific CD20-antigen, is commonly administered in RMDs. Considering that RTX is related to the development of hypogammaglobulinemia, it could increase the risk of infections [47]. In this context, it has been suggested that patients with low levels of IgG post-RTX may be at increased risk of serious infections since IgG levels are relevant for protective immunity. Infections seem to be more frequent during the first 3 months post-treatment, but they may occur anytime [47]. In addition to the immunosuppression caused by the hypogammaglobulinemia and the collapse of B and T cell immunity, RTX may also induce neutropenia, which may furtherly contribute to the risk of infections in these patients [47]. Finally, JAKis are approved for the treatment of RA, and they may enhance the risk of pneumonia and skin and soft tissues infections [48]. JAK are transmembrane proteins that mediate and amplify extracellular signals from growth factors and cytokines [43, 48, 49]. In accordance, JAKis exert a deleterious impact on adaptive immunity by decreasing CD4+T-cell expansion and Th1 and Th17 differentiation, among other effects [48–51]. Particularly, it was reported than the incidence rate for Herpes Zooster was higher than those estimated for other infectious events [49–51].

### Incidence and outcomes of COVID-19 in arthritis patients

RA and SpA are chronic inflammatory diseases, mainly affecting the joint, mediated by autoimmune and inflammatory process [52, 53].

More specifically, whereas RA is characterized by predominant peripheral synovial inflammation sustained by autoantibodies and B- and T-lymphocytes mechanisms, SpAs provide a main innate-immunity mediated pathogenesis leading to inflammation of synovial-entheseal structures and axial skeleton. SpA group includes ankylosing spondylitis (AS), psoriatic arthritis (PsA), reactive arthritis (ReA), enteropathic, and undifferentiated spondylarthritis (uSpA) [52, 53]. In patients with RA and SpA, it is recognized that the immunosuppressive therapy and the presence of comorbidities are associated with an increased risk of infections [54–57]. On these bases, a growing body of evidence described the characteristics of COVID-19 patients affected by RA [54–57]. In this context, it is unclear whether these patients may have an increased risk of infection or a higher risk of a severe course for COVID-19 [55–57]. On one hand, the host immunity could provide a strong immunological response adequate for virus clearance, whereas, on the other hand, an aberrant production of pro-inflammatory cytokines induced by the uncontrolled immune response may contribute to a development of cytokine storm syndrome worsening the prognosis of COVID-19 [58].

A recent nationwide Swedish study by Bower et al., comparing March-September 2020 inflammatory joint diseases cohort and 2015–2019 population referent, has evidenced that the risks of hospitalisation (0.5% vs 0.3% in their population referents), admission to intensive care (0.04% vs 0.03%) and death (0.10% vs 0.07%) due to COVID-19 were low in patients with RMDs and similar to general population [55]. This study has reported that overall absolute and excess risks for patients with inflammatory joint diseases are low and the level of risk increases are largely proportionate to those in the general population and explained by comorbidities [55]. Thus, this study showed a similar risk of severe COVID-19 in either RMDs or general population and suggested the negative prognostic impact of comorbidities in this context.

In a German registry, 468 patients with RMDs and a PCR-confirmed SARS-CoV-2 infection were studied [59]. Patients were stratified into three groups by using the hospitalization as indicator for a more severe course: (1) non-hospitalised patients, (2) hospitalised patients without the need for invasive ventilation, (3) hospitalised patients with the need for invasive ventilation. In this cohort, the most common disease was RA, affecting 225 patients (48%) and it was associated with a high rate of hospitalization. Out of those, 62 were hospitalised and 17 needed an invasive ventilation [59]. Out of 140 SpA patients with a PCR-confirmed SARS-CoV-2 infection showed that 20 were hospitalized and 5 needed an invasive ventilation [59]. In this cohort, when compared with RA, patients with SpA showed a lower risk of hospitalization (OR 0.46; 95% CI 0.23–0.91) [59]. In this cohort, only 38% did not have any other chronic condition, frequently concomitant comorbidities were registered in these patients. The most frequent one was high blood pressure, followed by obesity, cardiovascular disease (CVD), and diabetes. Regarding the administered therapy for the RMD, 193 patients were treated with csDMARD monotherapy, 167 patients with bDMARDs, and 145 patients with GCs, mostly low dose GCs of ≤ 5 mg/day. Older age, associated comorbidities, GCs at doses of > 5 mg/day, and moderate to high RMD disease activity were related to the rate of hospitalization [59].

In the COVID-19 Global Rheumatology Alliance physician-reported registry, a large collection of data about COVID-19 infection in RMD was reported, evaluating 600 patients from 40 countries, of whom 277 were hospitalized and 55 were deceased [60]. COVID-19 were predominately diagnosed through PCR testing. In this cohort, 230 patients were affected by RA which was the most represented RMD. A high percentage of these patients with RA (104 out of 230) were hospitalized. Results showed that 48 and 74 SpA and PsA patients, respectively, were diagnosed with COVID-19 predominately diagnosed through PCR testing [60]. A lower proportion of patients who were hospitalized had PsA and other SpA (8% and 6%, respectively) compared with those who were not (16% and 10%, respectively) [60]. Furthermore, a higher prevalence of comorbidities was also registered (hypertension 199 patients, lung disease 127 patients, diabetes 69 patients, CVD 63 patients and chronic renal insufficiency/end-stage renal disease 40 patients) [60]. The authors showed that older age and comorbidities were associated with the rate of hospitalization. On the contrary, the therapy with b/tsDMARD monotherapy, just before COVID-19 diagnosis, was significantly associated with a lower rate of admission to the hospital when compared with no DMARD therapy [60].

In addition, an observational French cohort described the characteristics of 694 patients with RMDs affected by COVID-19 [61]. The severity of COVID-19 was stratified as follows: (1) mild/ambulatory, (2) moderate/hospitalized out of intensive care unit, (3) severe/intensive care unit or deceased. SARS-CoV2 infection was mostly confirmed by PCR and/or specific antibodies. A large percentage of patients (492 out of 694) had at least one comorbidity (182 hypertension, 146 obesity, 99 respiratory disease, 85 CVD). Fifty-eight patients in this cohort died, resulting in an overall death rate of 8.3%, and of 22.6% in the hospitalized subgroup. In this study, 213 patients were affected by RA [61]. In this study the Authors described the characteristics of 165 patients with SpA and 70 patients with PsA [61]. Of those, great part of SpA patients showed mild infection [n. SpA patients: 135 (30.8%); n. PsA patients: 52 (11.9%)] and moderate infection (n. SpA patients: 25 (14.8%); n. PsA patients: 12 (7.1%)). SpA and PsA patients showing severe infection were 5 (5.8%) and 6 (6.9%), respectively. One patient with SpA and three patients with PsA died during the follow-up [61]. In a Spanish retrospective observational study, the Authors assessed patients with RMDs attending their rheumatology department and who were infected by SARS-CoV2 [62]. They described 62 patients with COVID-19, 42 were hospitalized and 20 were followed-up at home [62]. The most common RMD was RA in 20 affected patients (32%), of whom 3 patients died. Male gender, preexisting lung disease and treatment with GCs (≥ 5 mg per day) were associated with a more severe infection requiring hospitalization [62]. The Authors also described 16 patients with SpA/PsA, representing the 26% of the entire enrolled cohort with RMD and COVID-19 [62]. Eleven of those were hospitalized and 5 were followed-up at home. One of those died [62].

When compared with non-rheumatic hospitalized patients with SARS-COV2 infections, greater odds of hospitalization for patients with inflammatory arthropathies and connective tissue diseases were described by a Spanish group [63]. In a retrospective observational matched cohort study [63], 456 RMDs and non-rheumatic hospitalized patients with SARS-COV2 infection were described. Amongst them, 65 patients were affected by RA and 35 patients were affected by SpA [63]. This study also showed that comorbidities are associated with severe COVID-19 [63]. In this cohort, independent factors associated with severe COVID-19 were increased age, male sex, and having a connective tissue disease, but nor inflammatory arthritis nor previous immunosuppressive therapies were associated with severe COVID-19 [63].

In addition, an observational multicenter study (ReumaCoV Brasil register) assessed adult RMD patients with COVID-19 [64]. In this study, 334 patients with SARS-COV2 infection were included; the most common RMD was RA (28.4%). Hydroxychloroquine (34.9%), GCs (34.2%), MTX (20.1%), and TNFi (75/338; 22.2%) were the most common therapies [64]. This study showed that older age, use of GCs and cyclophosphamide were associated with unfavorable outcomes of SARS-CoV-2 infection. Conversely, TNFi appeared to have a protective role being associated with a reduction of the development of a more severe COVID-19 [64]. Finally, other small case series were available furtherly confirming these findings, as described in Table 1 [65, 66].

**Table 1:** Main studies focusing on Incidence, outcomes, and prognostic factors of COVID-19 in rheumatoid arthritis and spondiloarthritis patients

First author	Study design	Sample size	RA patients	SpA patients	PsA patients	Outcome of COVID-19 patients with inflammatory arthropathies	Prognostic factors
FAI2R /SFR/SNFMI/SOFREMIP/CRI/IMIDIATE consortium and contributors	Observational study	694	213	165	70	Severe COVID-19/hospitalization in RA: 29; SpA: 5; PsA: 6 Death in RA: 20; SpA: 1; PsA: 3	Older age Comorbidities Longer term of GCs
Gianfrancesco et al.	Observational study	600	225	48	74	Severe COVID-19/hospitalization in RA: 104; SpA: 16; PsA: 22 Death in the overall cohort including also CTD: 55	Older age Comorbidities Higher doses of GCs
Hasseli et al.	Retrospective observational study	468	146	125	*	Severe COVID-19/hospitalization in RA: 79; SpA: 20 severe COVID-19 Death in the overall cohort including also CTD: 19	Older age Comorbidities Treatment with GCs at doses > 5 mg/day Moderate to high RMD disease activity
Montero et al.	Retrospective observational study	62	20	16	*	Severe COVID-19/hospitalization in RA: 15; SpA: 11 Death in the overall cohort including also CTD: 10	Male Gender Preexisting lung disease Treatment with GC at dose > 5 mg/day
Pang et al.	Observational study	21	15	–	–	Severe COVID-19/hospitalization in RA: 15 0 death	Comorbidities
Cheng et al.	Observational study	5	4	–	–	5 hospitalised 0 death	Not reported
Marques et al.	Observational study	334	95	45	23	110 hospitalised 28 death	Age > 50 years Treatment with GCs and cyclophosphamide

GCs: Glucocorticoids; PsA: Psoriatic arthritis; RA: Rheumatoid arthritis; RMD: Rheumatic Diseases; SpA: Spondiloarthritis

*In these studies, PsA patients are included in the SpA group

### Arthritis and potential risk factors for severe COVID-19

In describing the characteristics of RA and SpA with COVID-19, many potential risk factors for a more severe disease have been suggested [59–63]. Similarly, to general population, age is considered a poor prognostic factor of COVID-19 for RA patients [67]. The possible age-associated shift in both innate and adaptive immune systems could reduce the capacity to deal infections and could contribute to the development of a chronic inflammatory state [68]. Moreover, a chronic low-grade inflammation called inflammaging is associated with immunosenescence, driven by a reduced ability to endure inflammatory triggers as well as an increased production of pro-inflammatory cytokines, acute phase proteins, and oxidative stressors, may lead to higher rates of infection and disease [68].

In addition, comorbidities, including chronic respiratory disease, CVD, diabetes, hypertension, obesity, and renal failure increased the risk for severe COVID-19, as reported in general population [55–62]. In fact, these comorbidities could modulate host‐viral or host‐immune system interactions favoring a more aggressive course of the disease [69]. Furthermore, the metabolic alterations produced by COVID-19 could decrease cardiorespiratory reserves following a possible stressor agent, enhancing the dysregulation of the immune system, and favoring a pro-thrombotic and pro-inflammatory state. The presence of diabetes would be considered one of the major risk factors for in‐hospital mortality increasing the risk of thromboembolism in COVID‐19 patients, disease severity, and mortality [69].

Antirheumatic drugs have been reported as not associated with increased risk of serious COVID-19 outcomes, although data accuracy was partial for certain drugs [55]. Moreover, some studies concerning the treatment of RA reported that the use of GCs was associated with a poorer outcome which may parallel with previous studies showing an increased risk of infection with higher doses of these drugs [31]. On the contrary, it has been suggested that no substantial risk was detected neither csDMARDS nor ts/bDMARD. mostly TNFis. Interestingly, the use of ts/bDMARD was also associated with a lower rate of complications during COVID-19 [61]. These findings are consistent with previous studies that found lower odds of hospitalization with ts/bDMARD monotherapy [60–63]. It could be possible that bDMARDs could reduce the high levels of cytokines, including IL-6 and TNF, associated with a more severe COVID-19, thus reducing the possibility to develop a CCS [61–63]. Of note, similar observations, about a beneficial effect of bDMARDs, were suggested on the risk of sepsis after serious infection or a fatal outcome [70].

Taking together these observations, to fully manage patients with RMDs and SARS-CoV-2 infection, it would be of crucial importance to understand how the characteristics of the underlying disease, associated comorbidities, and the use of immunotherapies could be associated with a more severe COVID-19 and a poorer outcome of affected patients [57, 60–63, 71]

### Incidence and outcomes of COVID-19 in patients affected by autoinflammatory disorders

Autoinflammatory diseases are caused by self-directed inflammation due to an alteration of innate immunity leading to episodic systemic inflammation attacks [72, 73]. Familial Mediterranean Fever (FMF) is the most common monogenic autoinflammatory diseases. It is an autosomal recessive disease characterized by recurrent systemic inflammatory attacks with fever and serositis [74]. Considering the possibility that COVID-19 could be associated with a CCS [58], the question of the susceptibility and severity of SARS-CoV-2 infection in patients displaying innate immunity disorders, such as FMF, has been raised. In this context, a survey on SARS-CoV-2 infection in patients with FMF followed in Paris area was conducted, including patients fulfilling the international FMF criteria and a genetic confirmed FMF diagnosis [63]. The authors collected the data of 342 patients, of whom 27 FMF patients (7.8%) were infected by SARS-CoV-2. Specifically, 7 patients were hospitalized, 6 required oxygen therapy, 3 developed acute respiratory distress syndrome and required admission to the intensive care unit, and 2 patients died. Severe SARS-CoV-2 infection occurred in patients with risk factors including advanced age, chronic kidney disease, hypertension, vascular disease, obesity, and lung dysfunction [63]. Based on these results, a dysfunction of the innate immune system of FMF could not appear to be a risk factor to develop a more severe COVID-19 [63]. In addition, a French national cohort study described 27 patients out of 694 with autoinflammatory disease affected by SARS-Cov2 infections [61]. Fifteen patients were affected by periodic fever syndromes, 7 patients by other autoinflammatory diseases, and 5 patients were diagnosed with adult onset Still’s disease (AOSD) [61]. Five patients experienced a severe COVID-19 and 4 died. Although of interest, these results should be cautiously interpreted due to the very low number of patients assessed by the authors [61]. Finally, a growing body of evidence points out that severe COVID-19 is characterized by an overlapping clinical picture with AOSD and macrophage activation syndrome (MAS), since these are characterized by fever, hyperferritinemia, and a hyper-inflammatory process with a massive release of pro-inflammatory cytokines [74, 75]. These findings would suggest that a hyperinflammatory lung injury, either in severe COVID-19 or MAS, would amplify the immune response leading to an overwhelming release of inflammatory mediators and the occurrence of a CCS [76].

### Appraisal of literature

As catastrophic COVID-19 pandemic continues to spread rapidly, many unresolved questions of how this virus would impact on RMDs and autoinflammatory disorders needs to be fully clarified [1–3]. Whether patients with RMDs could be at an increased risk of SARS- CoV-2 infection or severe COVID-19 disease remains to be entirely evaluated [14–16]. Incidence and outcomes of COVID-19 among RMDs and autoinflammatory disorders patients would appear to be consistent with general population affected by SARS-CoV-2 infection. In fact, patients with such diseases who are older and/or have comorbidities may have a higher odd of a more severe COVID-19 [55–57, 60, 63]. For the autoinflammatory disorders, the question is about a possible increased susceptibility to develop CCS in the context of COVID-19 since the prominent pathogenic involvement of the innate immune system in these diseases. However, the available data did not fully clarify this issue due to the low prevalence of these patients suggesting the need of further studies on this topic.

In addition, some data suggested that the real risk of developing severe forms of COVID-19 in patients with RMDs treated with csDMARD or bDMARDs might not increase [60–63]. It could be possible that these medications could reduce the high levels of proinflammatory cytokines thus possibly reducing the risk of a more severe disease during COVID-19 [60–63]. However, the risk of infections is reported to be consistent with such drugs [36–43] and further studies with a longer follow-up are needed to fully understand the real risk of these patients to develop a more severe SARS- CoV-2 infection. In this context, a possible theory could be that patients with RMDs could avoid risky behavior for infections being more aware for the possible risks associated with their immunosuppressive therapies. Furthermore, patients with RMDs may be associated with an increase frailty, which is defined as a syndrome characterized by a decrease of strength, endurance, reduced physiological function, and increased the individual’s vulnerability [77]. The chronic RMD inflammation may possibly contribute to the development of frailty associated with a characteristic pro-inflammatory T-lymphocyte phenotype as well as with an elevated concentration of IL-6 [78].

In addition, coagulation abnormalities are increasingly associated with a poor prognosis in COVID- [24]. Microthrombi of the lungs have been described in these patients [1, 24]. In fact, the activation of intravascular coagulation is a hallmark of organ injury in SARS-CoV-2 infection, which is mediated by pro-inflammatory cytokines [24, 73]. Furthermore, IL-1, IL-6, and TNF production and the endothelial cell dysfunction, associated with COVID-19, may play an important role in the thrombo-inflammatory processes ultimately impairing normal vascular haemostasis, fibrinolysis, and vessel wall permeability [79]. Thus, a high thrombotic risk of COVID-19 is clearly recognized which could be further increased by the concomitant RMDs [70, 79]. In fact, during these diseases, the pro-inflammatory milieu may induce a hyper-coagulation by an upregulation of procoagulant factors and a simultaneous downregulation of anticoagulant and fibrinolytic systems, thus leading to this clinical phenotype [79]. This risk is also associated with traditional CVD risk factors, such as an older age and diabetes, but also with the burden of the pro-inflammatory process over time and specific disease features [80, 81]. Taking together these observations, an accurate evaluation of the hyper-coagulation burden in the patients with RMDs affected by SARS-CoV2 infection is needed, since possibly associated with a worse prognosis.

Vaccination against SARS-CoV-2 is now available leading to a significant improvement in the management of COVID-19 [82, 83]. However, it could be matter of debate if the development of protective immunity could occur in patients with RMDs due to a possible impairment of the immune system by underlying inflammatory disease or therapies [82, 83]. Conflicting results are available in literature about this point [82, 83]. In a monocentric study enrolling 26 patients with immune-mediated inflammatory diseases (IMIDs), mainly RMDs, SARS-CoV-2 mRNA vaccines led to development of antibodies in immunosuppressed patients without considerable side effects [82]. Differently, another study on 84 patients with IMIDs, mostly RMDs, showed that immune responses against the SARS-CoV-2 were delayed and reduced [83]. In fact, the concomitant use of MTX appeared to adversely affect both humoral and cellular immune response to COVID-19 vaccines as shown in a more recent cohort study [84]. Taking together these observations, further studies are needed to entirely clarify this topic.

## Conclusions

The challenging situation of the SARS-CoV-2 infection is associated with the need of rapid access to the evolving evidence to close the gaps of knowledge which could make difficult to manage patients with chronic conditions. Since the early stages of the COVID-19 pandemic, many concerns arose regarding the potential consequences of SARS-CoV-2 infection in patients with RMDs or autoinflammatory disorders, given the potential negative effects of immunosuppressive drugs on viral clearance, thus speculating a possible increased COVID-19 severity and excess mortality in these patients. However, based on the current literature, available data would suggest that the real risk of developing severe forms of COVID-19 in patients with RMDs or autoinflammatory disorders treated with csDMARD or bDMARDS might not increase. Disease severity in COVID-19 is either similar or slightly higher among those with RMD, and risk appears to be largely associated with risk factors similar to the general population. In this context, it must be pointed out that further studies are likely to be needed to fully characterize these specific patient groups and associated SARS-CoV-2 infection.

## Data Availability

The reviewed data are included in the body of the article.
